# The Emerging Role of Bile Acids in the Pathogenesis of Inflammatory Bowel Disease

**DOI:** 10.3389/fimmu.2022.829525

**Published:** 2022-02-03

**Authors:** John P. Thomas, Dezso Modos, Simon M. Rushbrook, Nick Powell, Tamas Korcsmaros

**Affiliations:** ^1^ Gut Microbes and Health Programme, Quadram Bioscience, Norwich, United Kingdom; ^2^ Organisms and Ecosystem, Earlham Institute, Norwich, United Kingdom; ^3^ Department of Gastroenterology, Norfolk and Norwich University Hospital, Norwich, United Kingdom; ^4^ Department of Hepatology, University of East Anglia Medical School, Norwich, United Kingdom; ^5^ Division of Digestive Diseases, Imperial College London, London, United Kingdom

**Keywords:** bile acids, immunology and inflammation, gut metabolites, inflammatory bowel disease, Crohn’s disease, ulcerative colitis, gut microbiome

## Abstract

Inflammatory bowel disease (IBD) is a chronic immune-mediated inflammatory disorder of the gastrointestinal tract that arises due to complex interactions between host genetic risk factors, environmental factors, and a dysbiotic gut microbiota. Although metagenomic approaches have attempted to characterise the dysbiosis occurring in IBD, the precise mechanistic pathways interlinking the gut microbiota and the intestinal mucosa are still yet to be unravelled. To deconvolute these complex interactions, a more reductionist approach involving microbial metabolites has been suggested. Bile acids have emerged as a key class of microbiota-associated metabolites that are perturbed in IBD patients. In recent years, metabolomics studies have revealed a consistent defect in bile acid metabolism with an increase in primary bile acids and a reduction in secondary bile acids in IBD patients. This review explores the evolving evidence that specific bile acid metabolites interact with intestinal epithelial and immune cells to contribute to the inflammatory milieu seen in IBD. Furthermore, we summarise evidence linking bile acids with intracellular pathways that are known to be relevant in IBD including autophagy, apoptosis, and the inflammasome pathway. Finally, we discuss how novel experimental and bioinformatics approaches could further advance our understanding of the role of bile acids and inform novel therapeutic strategies in IBD.

## Background

Inflammatory Bowel Disease (IBD), comprising Ulcerative Colitis (UC) and Crohn’s disease (CD), is a complex immune-mediated inflammatory disorder of the gastrointestinal tract. It is a chronic, lifelong disease which causes significant morbidity (1). Despite recent advances in medical management, approximately 47% of patients with CD and 16% with UC require surgical intervention within 10 years of their diagnosis, highlighting the need for novel therapeutic strategies (2). IBD affects almost 7 million people worldwide, and the prevalence is forecasted to rise steeply in the decades ahead, particularly in newly industrialised countries (1). Although we now recognise that IBD arises due to complex interactions between genetic risk factors, an altered (dysbiotic) gut microbiota, and environmental factors, the precise mechanistic pathways interlinking these various facets of IBD pathogenesis are still largely unknown (3). Whilst metagenomic approaches have characterised the dysbiosis occurring in IBD patients (e.g. an increase in *Proteobacteria* and a decrease in *Firmicutes* species (4)) they have failed to demonstrate whether dysbiosis is a cause or effect of inflammation, and how this might disrupt the host-microbiota mutualistic relationship (5). Thus, a more reductionist approach has been suggested to deconvolute the complex interactions between the microbiota and the host (5). One such approach involves focusing on microbial metabolites—small molecules released or processed by the microbiota that affect a wide variety of host processes including intestinal epithelial barrier function, innate and adaptive host immunity, and host metabolism (6, 7). Three broad classes of microbiota-associated metabolites have emerged as having key roles in regulating intestinal homeostasis and inflammation in the context of IBD: short chain fatty acids (SCFAs), tryptophan metabolites, and bile acids (BAs) (6). In this review, we will evaluate the emerging evidence implicating BAs as important aetiological agents in the pathogenesis of IBD.

## The Physiology of Bile Acids

**Figure 1:**
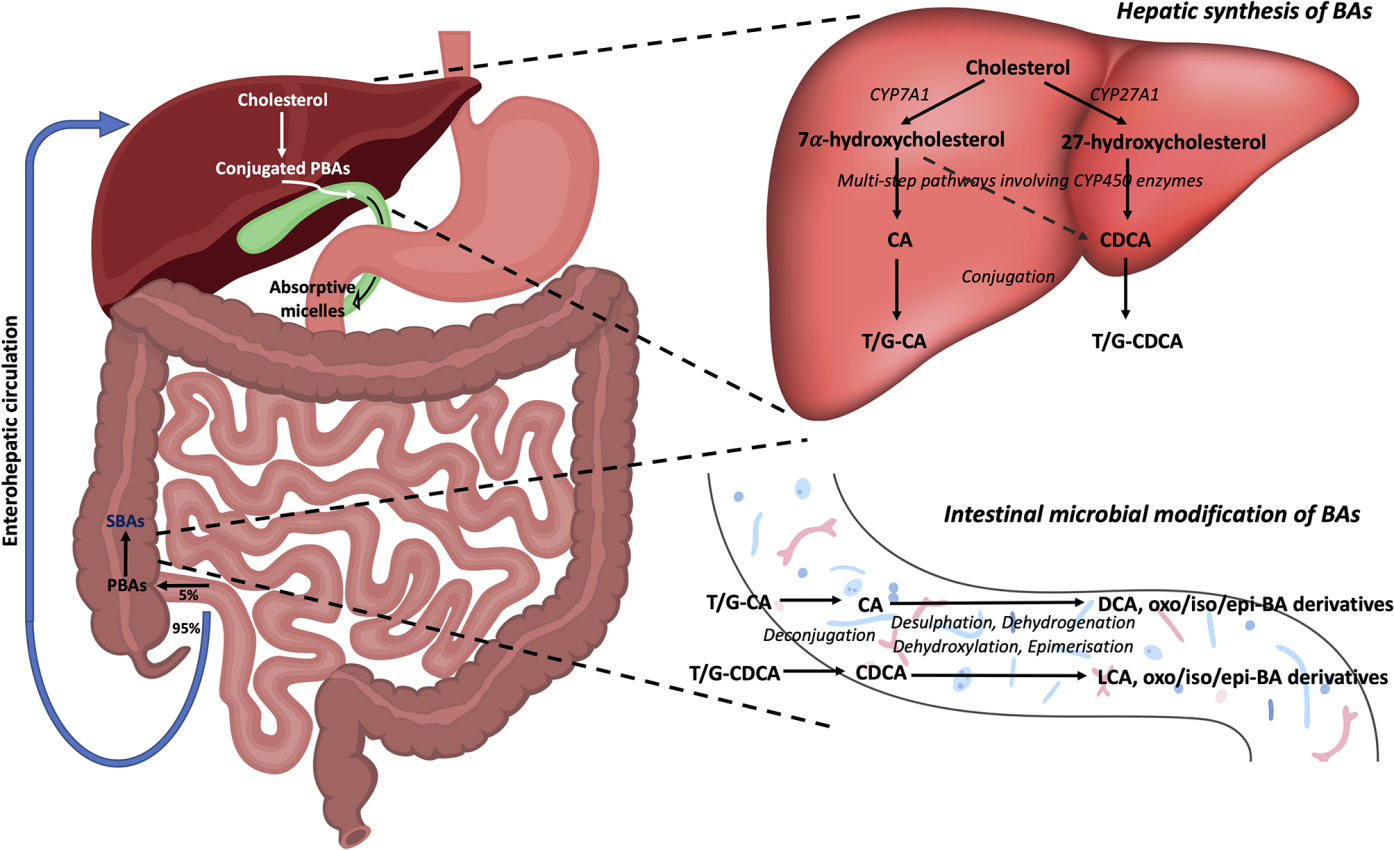
Bile acid synthesis, recirculation and microbial modifications in the gut. Bile acids are synthesised in the liver from cholesterol. In the classical pathway, cholesterol 7α-hydroxylase (CYP7A1) converts cholesterol to 7α-hydroxycholesterol which can then form either of the PBAs, CA and CDCA, through multistep pathways involving cytochrome P450 enzymes. In the alternative pathway, sterol-27-hydroxylase (CYP27A1) initiates the conversion of cholesterol to 27-hydroxycholesterol which mainly forms CDCA. These PBAs are then conjugated with either taurine (T) or glycine (G) to make them water soluble, before being released into bile where they form mixed micelles. As bile enters the duodenum *via* the ampulla of Vater, absorptive micelles are formed which facilitates the absorption of fatty acids, monoglycerides, fat soluble vitamins, and cholesterol in the small intestine. In the terminal ileum, 95% of conjugated BAs are reabsorbed through the enterohepatic circulation, whilst the remaining 5% enter the colon. Here, BAs undergo a number of chemical modifications by gut bacteria including deconjugation, desulphation, dehydrogenation, dehydroxylation, and epimerisation reactions to form the SBAs, LCA and DCA, and their oxo, iso, and epi-derivatives. PBAs, primary bile acids; SBAs, secondary bile acids; CA, cholic acid; CDCA, chenodeoxycholic acid; DCA, deoxycholic acid; LCA, lithocholic acid; T, taurine; G, glycine; CYP7A1, 7α-hydroxylase; CYP27A1, sterol-27-hydroxylase.

Primary bile acids (PBAs) in humans are 24-carbon atom molecules generated in pericentral hepatocytes from cholesterol through multiple steps that are catalysed by several enzymes including cytochrome P450 enzymes (8). This process can occur through either a classical or alternative/acidic pathway (**Figure 1**). During this process, cholesterol undergoes oxidative cleavage followed by addition of hydroxyl groups. As hydroxylation occurs only on one side of the molecule, PBAs have a hydrophilic surface and a hydrophobic surface, rendering them with amphipathic properties (9). Cholic acid (CA) and chenodeoxycholic acid (CDCA) are the two predominant PBAs found in humans. BAs are conjugated with glycine or taurine in hepatocytes prior to secretion into the bile canaliculi which increases their water solubility at acidic pH. In bile, conjugated PBAs form “excretory’’ mixed micelles with phosphatidylcholine and cholesterol, thus enabling the body to excrete cholesterol and other water-insoluble lipophilic molecules (9). Bile is released into the small intestine through the ampulla of Vater in the second part of the duodenum. In the small intestine, BAs form “absorptive” mixed micelles with fatty acids and monoglycerides which are products of dietary triglyceride lipolysis by pancreatic lipase, as well as fat soluble vitamins and cholesterol. BAs facilitate the reabsorption of these lipid molecules, which mostly occur in the jejunum (8). However, the conjugated BAs do not get absorbed and remain within the small intestine. In the terminal ileum, 95% of conjugated BAs that are secreted into the small intestine are reabsorbed through active transport and enter the liver *via* the portal vein (10, 11). This is known as the enterohepatic circulation of BAs.

The small proportion of BAs that remain in the intestines then undergo chemical alterations by colonic commensal bacteria including deconjugation, desulphation, dehydrogenation, dehydroxylation, and epimerisation (11). Deconjugation is mediated by bile salt hydrolase (BSH) enzymes which are widely distributed across gram-positive and gram-negative bacteria in the gut including species falling in the *Clostridium*, *Bifidobacterium*, *Bacteroides*, *Lactobacillus* and *Enterococcus* genera (12). Dehydroxylation at the carbon-7 position in deconjugated PBAs results in the generation of secondary bile acids (SBAs) (9). In humans, the major SBAs are lithocholic acid (LCA) and deoxycholic acid (DCA). Ursodeoxycholic acid (UDCA) is also another SBA in humans that is produced in small quantities by gut bacteria following epimerisation of the 7α-hydroxyl group of deconjugated CDCA (11). A range of *oxo*-, *epi*- and *iso*- derivatives of BAs can also be formed in the colon due to various dehydrogenation and epimerisation reactions by gut bacteria (13). These reactions in effect diversify the BA pool present in the luminal microenvironment, resulting in numerous BA metabolites that may act as signalling molecules between gut bacteria and the host (14). Whilst colonic bacteria are known to metabolise and produce these various BA metabolites, it is not yet clear which precise bacterial species or strains are required for this process [see recent review by Guzior and Quinn regarding bacteria responsible for microbial metabolism of BAs (13)]. Furthermore, the metabolic steps required for conversion of BAs by gut bacteria into the various BA derivatives are still not fully characterised. In fact, the complete metabolic pathway required to convert the PBAs, CA and CDCA, into the two major SBAs, DCA and LCA, was only recently mapped (15). This conversion was found to be mediated by a minimum set of 7α-dehydroxylating bile-acid-induced (bai) operon enzymes (BaiB, BaiCD, BaiA2, BaiE, BaiF, and BaiH) present within a limited range of *Clostridium* cluster XIVa species (e.g. *Lachnospiraceae* and *Ruminococcaceae* families), and also *Eubacterium* species.

It is important to note that dietary shifts can impart an additional layer of modulation on both gut microbial and BA composition (16, 17). A high-fat diet upregulates hepatic PBA synthesis, which results in increased PBA delivery to the colon where they undergo biochemical transformation to SBAs (18). Hence, individuals with high-fat diets have increased faecal SBAs. Studies have revealed that healthy rural Africans who consume a low-fat, high-fibre diet have lower levels of SBAs and 7α-dehydroxylating bacteria in their faeces, in comparison to healthy African Americans who consume a high-fat, lower-fibre diet (19). This correlated with the low incidence of colorectal (CRC) in rural Africans and high incidence of CRC in African Americans. Importantly, a switch in diet between these two populations resulted in higher levels of faecal SBAs and 7α-dehydroxylating bacteria in rural Africans, and lower levels of faecal SBAs and 7α-dehydroxylating bacteria in African Americans (20). The functional consequences of these changes due to a high-fat diet have been investigated in mice. Mice supplemented with a high-fat diet have a relative increase in the levels of the faecal SBA, DCA, in comparison to UDCA (21). This higher concentration of gut luminal DCA was found to disrupt epithelial barrier function in murine intestinal tissues (22). In another study, high-fat dietary supplementation in mice induced a shift in hepatic conjugation of BAs from glycine to taurine. This increased taurine-delivered sulphur content in the colon and in turn promoted the expansion of bile-tolerant microbes, in particular *Bilophila Wadsworthia* (23). This species was shown to induce Th1-mediated immune responses and colitis in *IL10^-/-^* mice.

**Figure 2:**
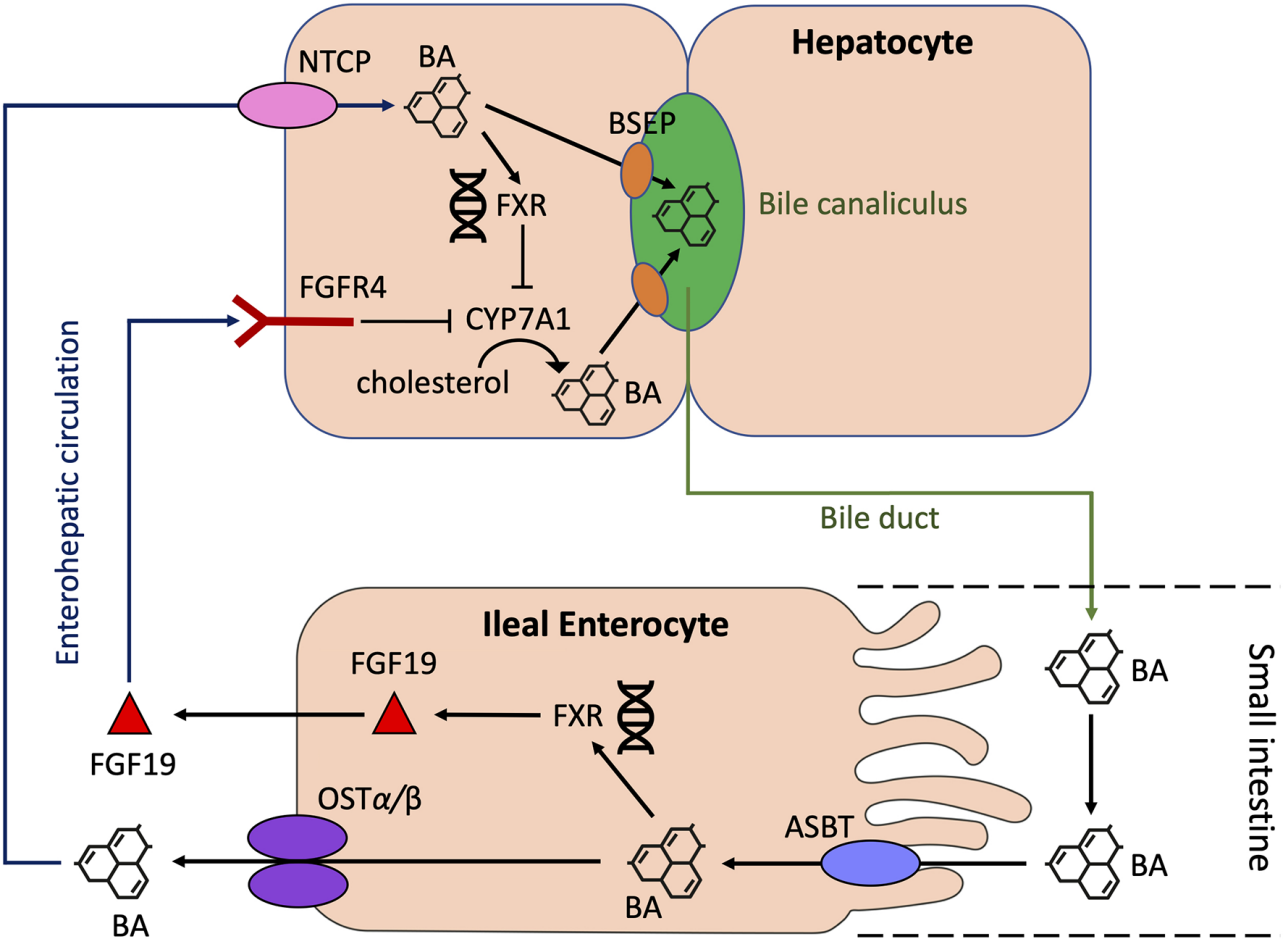
Regulation of BA synthesis by FXR and FGF19 in humans. Cholesterol 7α-hydroxylase (CYP7A1) initiates the conversion of cholesterol to PBAs through the classical pathway in hepatocytes. BAs are transported from hepatocytes into the bile canaliculus through the bile salt export pump (BSEP). BAs then enter the small intestine *via* the common bile duct. In the terminal ileum, enterocytes absorb BAs through the apical sodium-dependent bile salt transporter (ASBT). Here, BAs can activate the nuclear BA receptor, farnesoid X receptor (FXR), which results in the upregulation of fibroblast growth factor 19 (FGF19) and subsequent release of FGF19 into the portal circulation. BAs also enter the portal circulation *via* the organic solute transporter α/β (OST α/β) located in the basolateral membrane of enterocytes. BAs from the portal circulation are reabsorbed back into the liver *via* the Na^+^-taurocholate cotransporting polypeptide (NTCP). FGF19 also binds to fibroblast growth factor receptor 4 (FGFR4). Both these actions result in the inhibition of CYP7A1, thus resulting in the downregulation of BA synthesis. BA, bile acid; CYP7A1, cholesterol 7α-hydroxylase; BSEP, bile salt export pump; ASBT, apical sodium-dependent bile salt transporter; FXR, farnesoid X receptor; FGF19, fibroblast growth factor 19; NTCP, Na^+^-taurocholate cotransporting polypeptide; FGFR4, fibroblast growth factor receptor 4.

BAs can act on a range of nuclear and membrane receptors (**Table 1**) [reviewed in depth by Biagioli et al., 2021 (24)]. Nuclear receptors include Farnesoid X Receptor (FXR), Vitamin D Receptor (VDR), Retinoid Related Orphan Receptor (RORγt), Liver-X-Receptor (LXR), Pregnane X Receptor (PXR) and Constitutive Androstane Receptor (CAR). Cell membrane receptors include G-protein bile acid receptor 1 (GPBAR1) [also known as Takeda G protein-coupled receptor 5 (TGR5)], sphingosine-1-phosphate receptor 2 (S1PR2), cholinergic receptor muscarinic 2 and 3 (CHRM2, CHRM3), and the recently identified MAS Related GPR Family Member X4 (MRGPRX4) (25). BA synthesis is primarily regulated by the FXR. BA binding and activation of FXR on enterocytes, result in fibroblast growth factor 19 (FGF19) release in humans which acts on hepatocytes to inhibit BA synthesis (**Figure 2**) (11, 26). In mice, fibroblast growth factor 15 (FGF15) mediates this effect (18).

**Table 1:** Nuclear and membrane receptors of Bas.

Main BA receptor protein families	
Nuclear hormone receptors	Membrane-bound receptors
FXR	TGR5 (GPBAR1)
VDR	S1PR2
RORγt	CHRM2
CAR	CHRM3
LXR	MRGPRX4
PXR	

FXR, Farnesoid X Receptor; VXR, Vitamin D Receptor (VDR); RORγt, Retinoid Related Orphan Receptor-γt; LXR, Liver X Receptor; CAR, Constitutive Androstane Receptor; PXR, Pregnane X Receptor; TGR5, Takeda G protein-coupled receptor 5; GPBAR1, G-protein bile acid receptor 1; S1PR2, sphingosine-1-phosphate receptor 2; CHRM2, cholinergic receptor muscarinic 2; CHRM3, cholinergic receptor muscarinic 3; MRGPRX4, MAS Related GPR Family Member X4.

## Changes in Bile Acid Composition in IBD Patients

Recent landmark studies have characterised the general changes in BA composition that occur in IBD patients. Duboc et al. demonstrated that patients with colitis (n = 42: 12 CD, 30 UC) have impaired BA metabolism in active disease characterised by defective deconjugation, transformation to SBAs and desulphation, especially in active colitis (27). A recent comprehensive untargeted metabolomic and shotgun metagenomic profiling of stool samples from discovery (n = 155) and validation (n = 65) cohorts of IBD patients and non-IBD controls, revealed similar findings: out of >8000 measured metabolites, there was an increased abundance of PBAs such as CA and CDCA, with a corresponding reduction in SBAs (such as LCA and DCA) in IBD patients (28).

Furthermore, a major multi-omics profiling of 103 IBD patients (n = 38 UC, 65 CD) over one year revealed significant enrichment of the PBA CA and its glycine and taurine conjugates, in dysbiotic samples from CD patients in comparison to non-dysbiotic samples (29). In addition, the SBAs, LCA and DCA, were found to be reduced in dysbiotic samples from IBD patients. Complementary findings were also reported by Sinha et al, in which there was a significant reduction in the SBAs (LCA and DCA) in the ileal pouches of colectomy-treated UC patients (n=17), in comparison with pouches of colectomy-treated Familial Adenomatous Polyposis (FAP) patients (30).

Jacobs et al. have reported similar findings in paediatric IBD patients, where they observed increased PBAs and/or their conjugated forms (CA, CDCA sulphate, 7-sulphoCA) as well as less well-known BAs including 7-ketodeoxycholic acid and 3-sulfodeoxycholic acid in IBD patients (31). Corresponding observations were noted by Wang et al. who found a significant reduction in SBAs and unconjugated BAs in a cohort of 29 paediatric CD patients in comparison to 20 healthy subjects (32). These changes in the BA pool in CD patients correlated with reduced abundances of bacterial genera that contained BSH (*Bacteroides*, *Bifidobacteria*, *Clostridium*, and *Lactobacillus*) and 7α-dehydroxylation enzymes (*Clostridium* and *Eubacterium*).

Overall, these findings suggest a consistent defect in BA metabolism with a resultant increase in PBAs (and/or their conjugated forms) and reduction in SBAs in IBD patients (6, 24, 33). Interestingly, recent studies have demonstrated that shifts in the BA pool may also predict therapeutic response, further strengthening the idea that BAs may play an important role in the pathogenesis of IBD. Ding et al. found that CD patients that responded to anti-TNF therapy had greater serum levels of a SBA (DCA) and certain tertiary BAs, whilst those who did not respond had higher levels of serum unconjugated PBAs (i.e. CA and CDCA) (34). Similarly, Lee et al. noted that lower levels of faecal DCA was associated with higher levels of intestinal inflammation in IBD patients (35). In addition, the authors found that patients whose gut microbiota possessed the aforementioned set of 7α/β-dehydroxylation bai operon enzymes required for conversion of PBAs to SBAs, had a higher abundance of faecal SBAs and were more likely to respond to anti-cytokine therapies.

Further compelling evidence for the role of BAs in regulating intestinal inflammation come from studies in patients with *Clostridium difficile* infection (CDI) - a major cause of infective gastroenteritis worldwide (36). Whilst recent antibiotic exposure, immunosuppression and older age are established risk factors for CDI, the underlying mechanistic pathways contributing to its pathogenesis have only become more apparent in recent years. A variety of experimental approaches have revealed that perturbations to the gut microbiome resulting in loss of gut microbial species containing BSH and 7α-dehydroxylation enzymes, and subsequent increases in conjugated PBAs (in particular taurocholic acid) and reductions in SBAs, promote *Clostridium difficile* spore germination and growth in the gut (37–39). Furthermore, faecal microbiota transplantation (FMT) which is a highly efficacious therapy for recurrent or refractory CDI (rCDI), is also dependent on BA metabolism. Studies have shown that the efficacy of FMT in rCDI is in part determined by its ability to restore BSH activity and SBA biosynthesis in the gut microbiome (40).

Recent randomised controlled trials (RCTs) and prospective studies have also demonstrated that FMT is an effective approach for inducing remission in UC (41–44). Interestingly, UC patients who achieved remission after FMT were found to have enrichment of *Eubacterium hallii* and the *Firmicute*, *Roseburia inulivorans*, with increased levels of faecal SCFAs and SBAs (45). Although preliminary studies suggest that FMT may also be an effective therapy in CD, large RCTs are currently lacking (46). However, exclusive enteral nutrition (EEN) is an alternative microbiota-modulating therapy that is highly efficacious and well-established for inducing remission in paediatric CD (47). Again, BA metabolism appears to be an important determinant of EEN therapeutic response as CD patients that had increased levels of faecal PBAs prior to receiving EEN were less likely to achieve sustained remission (48). However, those who achieved sustained remission had SBA-dominant faecal profiles before, during, and after EEN. In addition, EEN has been found to alter the faecal BA profile in paediatric CD patients with increased SBAs and reduced PBAs and conjugated BAs (49). Given these findings, SBA supplementation could be a potential strategy for inducing or maintaining remission in IBD patients. Pre-clinical studies have already demonstrated that supplementation with the SBA, UDCA, in mouse models of colitis reduces inflammation and partially resolves dysbiosis (50, 51). Previous clinical trials have supplemented UDCA in UC patients with primary sclerosing cholangitis (PSC) but for the purpose of evaluating whether it can lower the risk of developing CRC (52). Whilst, UDCA was shown to be chemopreventive in UC PSC patients (53, 54), the effect of SBA supplementation in IBD patients for ameliorating inflammation has yet to be determined. Of interest, Sinha and Habtezion are currently leading a clinical trial to investigate whether UDCA may reduce inflammation and improve quality of life in UC patients who develop pouchitis following total colectomy (clinicaltrials.gov identifier: NCT03724175).

Although these studies have been helpful in gaining initial insights into the changes occurring in the gut BA composition of IBD patients, further work is required to address important unanswered questions - in particular, characterising the functional consequences this may have on the regulation of intestinal inflammatory responses. However, future pre-clinical and clinical studies must take into account a number of important factors that may influence the metabolome of IBD patients. This includes distinguishing between the various sub-phenotypes of IBD (e.g. proctitis and pancolitis in UC), ensuring patients with subclinical PSC are not included, capturing dietary history, and accurately charting IBD disease activity and exposure to therapies when collecting data from patients. Studies should also aim to characterise the wider repertoire of BA metabolites present within the human gut rather than just the handful of BAs that are commonly evaluated, as these lesser-known BA metabolites may also impart important effects on the gut mucosa and immune response. A recent metabolomics study demonstrated the importance of this, as the investigators identified three novel BAs (phenylalanocholic acid, tyrosocholic acid, and leucocholic acid) in mice and found these to be enriched in faecal samples from IBD patients (55).

## Immune Regulatory Effects of Bile Acids on the Intestinal Mucosa

Emerging evidence indicates that the shift in PBA and SBA composition detected in IBD patients may contribute to pro-inflammatory intestinal mucosal responses through differential effects on epithelial and immune cells compared to the normal state. PBAs and SBAs including *oxo* and *iso BA* derivatives, act on a variety of BA-activated receptors which are highly expressed in intestinal tissues. These receptors have been found to be expressed on both epithelial cells of the gut as well as immune cells including type 3 innate lymphoid cells (ILCs), Th17 cells, dendritic cells (DCs), natural killer cells (NKCs), and monocytes/macrophages, mainly from mice studies [as detailed in the following reviews (11, 24, 56, 57)]. Interestingly, single nucleotide polymorphisms (rs3863377, rs7138843, rs56163822, rs35724, rs10860603*)* in the non-coding region of the *FXR* gene have also been found to be associated with IBD (58).

## Bile Acids and Intestinal Immune Cells

**Figure 3:**
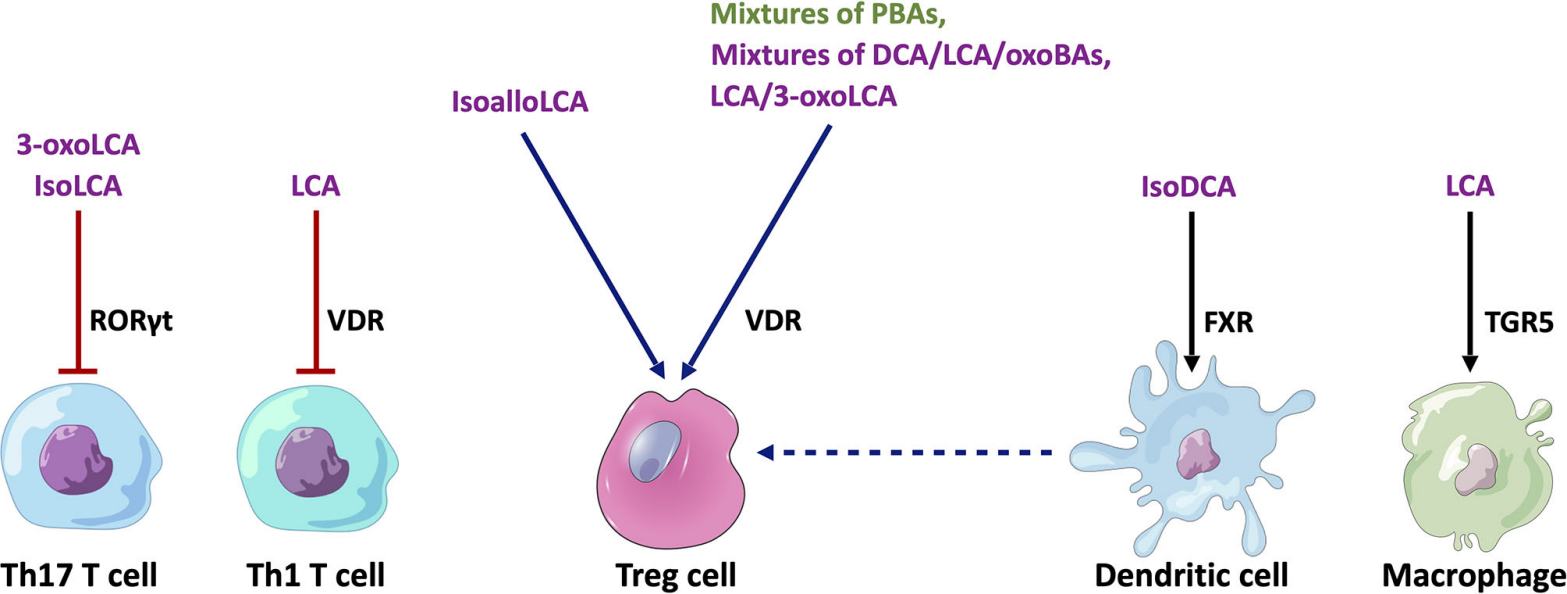
Cell type-specific effects of BAs on immune cell populations relevant to IBD. Mixtures of PBAs (CA/UDCA, CDCA/UDCA or CA/CDA/UDCA) or SBAs (DCA/LCA/3-oxoCA/3-oxoLCA/7-oxoCA/7-oxoCDCA/12-oxoCA/12-oxoDCA or LCA/3-oxo-LCA) have been found to stimulate RORγt^+^ FOXP3^+^ Treg cells by acting on the VDR in mice (N.B. In mice, UDCA is considered to be a PBA). IsoalloLCA can stimulate the differentiation of FOXP3^+^ Treg cells through an epigenetic pathway. 3-oxoLCA and isoLCA inhibit the differentiation of Th17 T cells by acting on the RORγt receptor, whilst LCA inhibits Th1 T cell differentiation through a VDR-dependent mechanism. IsoDCA induces an anti-inflammatory profile in DCs by acting on the FXR, which leads to the promotion of RORγt^+^ FOXP3^+^ Treg cells. Similarly, LCA induces an anti-inflammatory phenotype in macrophages by activating TGR5. Purple text - SBAs, Green text - PBAs, Blue arrows - stimulatory interactions, Red arrow - inhibitory interaction, Black arrow - anti-inflammatory effect. PBAs, primary bile acids; SBAs, secondary bile acids; DCA, deoxycholic acid; LCA, lithocholic acid; RORγt, Retinoic acid-related orphan receptor gamma t; VDR, vitamin D receptor; FXR, farnesoid X receptor; TGR5, Takeda G protein-coupled receptor 5.

A number of recent studies using mouse models have provided initial insights into the functional role of BAs in shaping immune responses in IBD involving various immune cell populations (**Figure 3**).

### Macrophages

Sinha et al. showed that rectal SBA (i.e. LCA) administration ameliorated inflammation in dextran sodium sulphate (DSS) colitis, acute trinitrobenzenesulfonic acid (TNBS) colitis, and chronic CD45RB^hi^ T cell transfer colitis in mice (30). In this study, LCA exposure decreased the expression of several proinflammatory cytokines (CXCL10, IL17A, TNFα, CCL5) in distal colonic tissues from DSS colitis-inflamed mice. This protective effect of LCA in DSS-colitis was dependent on TGR5 receptor expression in immune cells rather than in epithelial cells. Earlier work has shown that macrophages may be the key immune cell type that is targeted by LCA (and DCA) *via* TGR5. Yoneno et al. demonstrated that TGR5 is expressed highly in a certain subset of peripheral blood-derived macrophages, which closely resemble pro-inflammatory intestinal CD14+ macrophages that are implicated in IBD (59). In this macrophage subtype, LCA and DCA inhibited TNFα production following exposure to commensal bacterial antigens or lipopolysaccharide. This effect was shown to be likely mediated through the TGR5-cAMP pathway, leading to phosphorylation of c-Fos and subsequent binding of NFκB p65. Another study has demonstrated that TGR5 activation may promote the shift of M1 pro-inflammatory colonic macrophages towards an anti-inflammatory M2 phenotype through a different mechanism (60). TGR5 activation was shown to activate protein kinase A (PKA), resulting in phosphorylation of the transcription factor, cAMP response element-binding protein (CREB). Phosphorylated CREB subsequently inhibited the transcription of pro-inflammatory cytokines such as TNFα and IL1β, and induced the transcription of anti-inflammatory cytokines such as IL10. In this way, Biagioli et al. revealed that TGR5 activation could ameliorate inflammation in TNBS and oxazolone colitis models (60).

### T Regulatory Cells

There is compelling evidence that certain BA metabolites are important regulators of colonic T regulatory (Treg) cells. Song et al. revealed that the specific combinations of BA metabolites in the murine gut regulate a distinct FOXP3^+^ T reg cell population within the colon expressing the transcription factor RORγt by acting on the VDR (61). The investigators supplemented the feeding water of minimal-diet specific pathogen-free (SPF) mice with mixtures of murine PBAs and SBAs. They found that particular combinations of PBAs (CA/UDCA), CDCA/UDCA or CA/CDA/UDCA) or SBAs (DCA/LCA/3-oxoCA/3-oxoLCA/7-oxoCA/7-oxoCDCA/12-oxoCA/12-oxoDCA or LCA/3-oxo-LCA) significantly increased the number of colonic RORγt^+^ FOXP3^+^ Treg cells in these mice. In addition, supplementation with mixtures of the three murine PBAs or eight SBAs provided resistance to the onset of colitis following dextran sulphate sodium (DSS) challenge. Deficiency of the VDR resulted in the failure of BA supplementation to restore colonic RORγt^+^ FOXP3^+^ Treg cells in these mice and also worsened colitis upon DSS challenge.

In a separate study, Campbell et al. showed that the SBA, isoDCA (3β-hydroxydeoxycholic acid), increased induction of colonic RORγt^+^ FOXP3^+^ Tregs by acting on dendritic cells (DCs) to diminish their immunostimulatory properties through the FXR (62). Hang et al. also demonstrated that another SBA derivative, isoalloLCA, substantially affected the differentiation of FOXP3^+^ Treg, using a screen of 30 BAs (both PBAs and SBAs) in mice (63). IsoalloLCA upregulated FOXP3^+^ Treg cell differentiation by enhancing mitochondrial reactive oxygen species (mtROS) production and through an epigenetic mechanism which requires TGFβ-induced signalling. Recently, Li et al. further characterised this molecular mechanism (64). They revealed that this epigenetic change involved induction of an open chromatin region at the *Foxp3* gene promoter site, resulting in the binding of the nuclear hormone receptor NR4A1 at the *Foxp3* locus. This led to increased *Foxp3* transcription through a mechanism dependent on the enhancer conserved non-coding sequence 3 (CNS3). Interestingly, the investigators also identified that levels of isoalloLCA and the biosynthetic gene cluster responsible for its generation that is abundant in Bacteroidetes species, are significantly reduced in IBD patients. These studies suggest that regulation of Tregs by BA metabolites may be a key mechanism for immune tolerance in the human gut.

### Effector T Cells

BA metabolites have also been found to regulate the differentiation of effector T cell populations. Pols et al. revealed that physiological concentrations of unconjugated LCA could inhibit the activation of primary human and mouse CD4^+^ Th1 cells through a VDR-dependent mechanism, resulting in decreased TNFα and INFγ production (65). Hang et al. demonstrated that 3-oxoLCA inhibited Th17 cell differentiation by physically interacting with RORγt and inhibiting its transcriptional activity in mice (63). Hence, administration of 3-oxoLCA in mice led to a reduction in the differentiation of Th17 cells in the intestinal lamina propria. Recently, Paik et al, showed that like 3-oxoLCA, isoLCA also inhibits Th17 differentiation by inhibiting RORγt. Using a high-throughput screen of human isolates, they identified human gut bacteria and their corresponding enzymes required to convert LCA to 3-oxoLCA and isoLCA (66). 12 bacterial genera (*Adlercreutzia, Bifidobacterium, Enterocloster, Clostridium, Collinsella, Eggerthella, Gordonibacter, Monoglobus, Peptoniphilus, Phocea, Raoultibacter, Mediterraneibacter*) in the human gut microbiome were found to harbour the enzymes required for 3-oxoLCA and isoLCA synthesis. Multi-omics analyses of two IBD registries revealed that 3-oxoLCA and isoLCA, as well as the bacterial 3α-hydroxysteroid dehydrogenase genes (3α-HSDH) responsible for the production of these metabolites, were significantly reduced in patients with IBD (66). Furthermore, levels of these BAs were found to inversely correlate with the expression of pro-inflammatory Th17 cell-associated and IL17 pathway-related genes in an IBD patient cohort.

## Bile Acids and Intestinal Epithelial Cells

Initial studies in this area focused on the pro/anti-carcinogenic effects of BAs in the setting of colorectal cancer. DCA and CDCA, but not CA or UDCA, have been shown to induce apoptosis in colorectal cancer cell lines (67, 68). DCA has also been demonstrated to exert genotoxic effects through oxidative stress and suppression of p53 response to DNA damage (69, 70). *In vivo* models of cancer have also shown that DCA may have pro-carcinogenic roles, whilst UDCA may exert anti-carcinogenic effects. In the azoxymethane mouse model of colorectal carcinogenesis, administration of DCA significantly increased K-ras mutations and the incidence of colonic tumours (71), whilst UDCA was shown to suppress the development of colorectal cancers with K-ras mutations (72). The protective effects of UDCA have been extended to humans through clinical trials. UDCA lowered cancer mortality in UC patients with primary sclerosing cholangitis (UC-PSC) (73) (although not a consistent finding) and significantly reduced recurrence of high-grade dysplastic tumours in patients following removal of colorectal adenoma (74). BA receptor expression may also modulate the anti-carcinogenic effects of certain BAs, as *FXR* expression was found to inversely correlate with the risk of colorectal cancer in UC-PSC patients (75).

**Figure 4:**
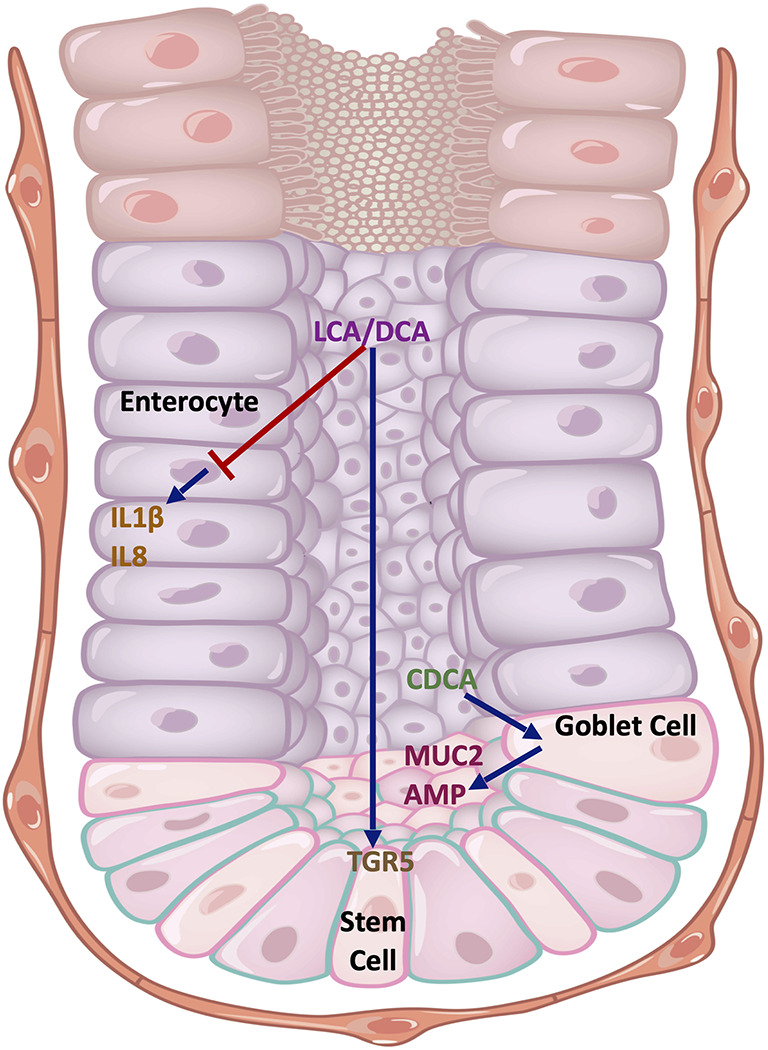
Cell type-specific effects of BAs on the intestinal epithelium. The SBAs, LCA and DCA, inhibit the production of pro-inflammatory cytokines IL1β and IL8 in Caco-2 cells that resemble enterocytes. They also promote stem cell growth in the intestinal crypts by acting on TGR5. CDCA acts on goblet cells to promote MUC2 expression and antimicrobial peptide (AMP) release. Key: Blue arrows - stimulatory interactions, Red arrow - inhibitory interaction. DCA, deoxycholic acid; LCA, lithocholic acid; CDCA, chenodeoxycholic acid; MUC2, mucin 2; AMP, antimicrobial peptides; TGR5, Takeda G protein-coupled receptor 5; IL1β, interleukin 1-beta; IL18, interleukin 18.

However, the effect of BAs on intestinal epithelial regulation of inflammation is less well characterised (**Figure 4**). Duboc et al. exposed the Caco-2 human colon adenocarcinoma cell line to BAs, and demonstrated that SBAs (DCA and LCA), but not PBAs, inhibited IL1β-induced IL8 secretion (27). Recently, it was demonstrated that SBAs (LCA and DCA) promote intestinal epithelial regeneration in mice by acting on the TGR5 receptor in intestinal stem cells (ISCs) (76). Loss of TGR5 function in ISCs led to an accelerated onset and progression of DSS-induced colitis, together with an impaired regenerative capacity. Similar protective effects of SBAs (UDCA and LCA) in DSS colitis were found in a separate study through promotion of epithelial barrier function (77). Tremblay et al, however, demonstrated homeostatic effects with the PBA, CDCA, on murine ileal explants (78). In this study, mice supplemented with CDCA showed elevated levels of antimicrobial peptides in the ileal epithelium, most likely from a variety of cellular sources including Paneth cells and goblet cells. In addition, there was an upregulation in the expression of *Muc2* from goblet cells.

In summary, studies predominantly in mice have demonstrated that particular SBAs exert anti-inflammatory effects on the intestinal mucosa, although certain PBAs or combinations of PBAs may impart protective effects too. These studies reveal cell type- and receptor- specific effects of BAs on intestinal homeostasis and inflammation. Thus, alterations to the gut BA composition have potential to contribute to dysregulated intestinal inflammatory responses through cell type-specific effects.

## Potential IBD-Relevant Cellular Signaling Pathways Mediating Effects of BAs

Based on the current literature, a number of key intracellular pathways may mediate the effects of BAs in the intestines that are relevant to IBD. These include the inflammasome pathway, apoptosis, and autophagy.

### Inflammasome Pathway

In recent years, inflammasomes have emerged as an important regulator of mucosal immunity as well as host responses to certain important classes of microbial-associated metabolites, such as BAs and SCFAs, in the context of IBD (79).

Inflammasomes are cytosolic multiprotein complexes which orchestrate inflammatory response to pathogenic and host danger signals (80). They are present in a variety of cell types including immune cells, haematopoietic cells and epithelial cells (81). Since their original description in 2002, different types of inflammasome complexes have been identified, including NLRP1, NLRP3, NLRC4, and AIM2, which differ in their protein components. Of these, the NLRP3 inflammasome complex is the best characterised, and it has also been implicated in IBD pathogenesis. The NLRP3 inflammasome consists of three major protein components - the sensor NLRP3 protein, the adaptor apoptosis-associated speck-like protein containing a C-terminal caspase recruitment domain (ASC), and procaspase-1 (80).

Various IBD genetic susceptibility variants have been linked to the NLRP3 inflammasome-IL-1β/18 axis including *NFκB, CARD8, IL18, IL1β* and the *NLRP3* gene itself (82). Interestingly, some of these polymorphisms have been shown to predict response to anti-TNF therapy (83). Experimental studies in mice and human studies have revealed varying roles of the NLRP3 inflammasome in the pathogenesis of IBD. Some studies have suggested a pro-inflammatory effect. For instance, mice deficient in NLRP3 (*Nlrp3^-/-^*) have reduced inflammation in two IBD model systems (DSS and TNBS colitis) (84). Furthermore, an upregulation of NLRP3 inflammasome components with increased mRNA expression of *NLRP3, IL1β, ASC* and *Caspase-1* has been observed in colonic biopsies in both CD and UC patients, which was correlated with increased disease activity (85). However, other studies indicate an anti-inflammatory effect. For instance, mice with *Nlrp3, Asc*, or *Caspase-1* deficiency have been found to exhibit more severe experimental (DSS-induced) colitis and decreased intestinal epithelial integrity in some studies (86). Similarly, genetically modified mice carrying the *Nlrp3* R258W mutation, which induces hyperactivation of NLRP3 inflammasome, were strongly resistant to experimental colitis (87).

Guo et al. have previously demonstrated that BAs can regulate the activity of the NLRP3 inflammasome (88). In this study, they revealed BAs (both primary and secondary) inhibit NLRP3 inflammasome activation through post-translational modifications. They showed that BAs (in particular the SBA, LCA) binds to TGR5 on macrophages, which results in adenylate cyclase and PKA activation. PKA was found to then directly phosphorylate NLRP3 at Ser291, leading to enhanced ubiquitination of NLRP3. This prevented NLRP3 from assembling with the other inflammasome components, ASC and pro-caspase 1, resulting in diminished IL1β production from macrophages. Similar findings have been observed in the liver whereby TGR5 activation by the SBA, DCA, resulted in attenuation of macrophage-related inflammation and promoted anti-inflammatory macrophage M2 polarisation (89). However, contrasting results have been observed by Zhao et al. They demonstrated that DCA could activate the NLRP3 inflammasome partly by acting on the sphingosine-1-phosphate receptor 2 (S1PR2) receptor and stimulating ERK-dependent cathepsin B release (90). This triggered IL1β release from macrophages. Colorectal administration of DCA enhanced IL1β levels in murine colonic tissue and exacerbated DSS colitis, which was ameliorated following S1PR2 inhibition, NLRP3 inhibition, or macrophage depletion. The reasons for these divergent findings are not clear but may indicate receptor- and cell type-specific effects of BAs on the NLRP3 inflammasome.

### Apoptosis

The intestinal epithelial barrier is maintained through tightly regulated processes of stem cell renewal, proliferation, differentiation, migration, cell death *via* apoptosis and cell shedding. Failure to finely coordinate these processes can lead to epithelial barrier dysfunction and increased barrier permeability, which are characteristic features of IBD (91). As such, dysregulation of apoptosis may have a key role in the pathogenesis of IBD as evidenced by animal models. In mice, impaired NFκB signalling can lead to apoptosis of colonic epithelial cells and reduced antimicrobial peptide release, which results in translocation of bacteria into the mucosa and increased susceptibility to DSS colitis (92). Similarly, conditional *Stat3* knockout in intestinal epithelial cells of mice, can also lead to increased susceptibility to experimental colitis and upregulation of apoptosis (93). Analogous increases in levels of apoptosis have been observed in the intestinal epithelium of patients with IBD (93–95).

Apoptosis may also be important for mediating therapeutic response in IBD. In the lamina propria, T cells have been found to be resistant to apoptosis in patients with CD (96, 97). This T cell resistance along with modulation of apoptosis in other lamina propria mononuclear cells has been linked to the efficacy of anti-TNF antibodies in IBD patients (98–100).

Interestingly, some studies have reported that BAs can influence apoptosis in colonic epithelial cells. Exposure of UDCA or LCA was found to ameliorate DSS-colitis in mice partly through the inhibition of epithelial apoptosis and promotion of epithelial barrier function (77). However, it has also been demonstrated that persistent exposure to hydrophobic BAs such as DCA in the colonic epithelium can lead to oxidative stress and genomic instability, and also select for a population of apoptosis-resistant epithelial cells (101, 102). This may increase the risk of colonic carcinogenesis.

### Autophagy

Macroautophagy (herein referred to as autophagy) is another critical cellular process that is perturbed in IBD. Autophagy is a catabolic process by which specific or damaged intracellular cytosolic proteins and organelles are engulfed and carried within a double-membrane vesicle called the autophagosome to the lysosome for degradation (103, 104). Products of this degradation process can then be recycled for maintaining cellular metabolism and function. Autophagy has a basal level of activity in normal physiological conditions. Autophagy plays a pivotal role in removing and recycling damaged organelles and aggregated proteins to maintain intracellular quality control (105). However, autophagy can also be induced in times of stress where it acts as a survival response to starvation and a number of cellular stresses including genotoxic stress, metabolic stress, and infection.

Autophagy was first recognised as a key player in IBD pathogenesis when it was discovered that one of the most common CD-associated single nucleotide polymorphisms occurs within the coding region of *ATG16L1* (106). Since then, several autophagy-related genes (e.g. *NOD2, IRGM, LRKK2, DAP1*) have been found to be associated with both CD and UC (107). Several landmark studies in the past decade have unravelled the key role of autophagy in IBD. Mice with conditional deletion of *Atg16l1* in intestinal epithelial cells (IECs) were found to display evidence of endoplasmic reticulum (ER) stress in the base of intestinal crypts (108), similar to that seen in patients with CD who harbour the risk variant (109). In addition, deletion of *Xbp1* in these mice resulted in discontinuous, transmural ileitis with deep fissures in the small bowel akin to that seen in CD patients.

A few studies have reported that BAs may affect autophagy in a variety of cell types including epithelial cells, immune cells, hepatocytes, adipose tissue, and pancreatic cells. Payne et al. revealed that high-concentrations of DCA was cytotoxic to colonic epithelial cells (NCM-460) through the activation of apoptosis secondary to ER stress, DNA damage, and mitochondrial stress (110). However, DCA could also induce autophagy as a pro-survival stress response through the generation of reactive oxygen species (ROS) and could prevent cells from undergoing DCA-induced apoptosis (but not DCA-induced necrosis). This was evidenced by increased formation of autophagosomes, levels of LC3-I and LC3-II, and expression of beclin-1 following DCA exposure. In addition, pre-treatment with rapamycin (autophagy activator) and 3-methyladenine (autophagy inhibitor), resulted in significantly decreased and increased cell death, respectively. The authors also found similar protective effects with autophagy upregulation in an apoptosis-resistant colonic cancer cell line (HCT-116) that had been developed following persistent exposure to increasing concentrations of DCA. Thus, the balance between autophagy and apoptosis may be critical for determining the fate of colonic epithelial cells exposed to DCA. This effect however may be BA specific, as another report suggested that whilst DCA could upregulate autophagy in T84 colonic adenocarcinoma cells, UDCA had little effect on autophagy (111). Furthermore, Wammers et al. revealed using transcriptomic analysis that exposure of taurolithocholic acid in contrast suppressed autophagy along with other functions such as phagocytosis and pro-inflammatory pathways (112).

Interestingly, hepatic autophagy may also determine BA signalling in the gut. Yan et al. found that hepatic autophagy deficiency (*Atg5Δhep*) induces liver injury in mice and also altered the BA pool with a reduction in tauro-conjugated BAs and an increase in unconjugated BA (113). This caused significant perturbations to the gut microbiome with a rise in BA-metabolising microbiota. These changes led to the upregulation of FXR signalling in ileal epithelial cells (as FXR has a higher affinity towards unconjugated BAs), resulting in increased FGF15 levels. FGF15 signalling had a protective effect against liver injury. Thus, deficient hepatic autophagy can impart important changes to BA signalling in the gut which acts as an adaptive mechanism to protect against liver injury.

## Future Perspectives

The current evidence demonstrates that BAs are an important class of microbial-associated metabolites that shape inflammatory responses in the gut and can interact with a number of critical cellular pathways relevant in IBD. These studies have largely taken place using mouse models. Whilst useful from a mechanistic perspective, humans and mice have significant differences in their BA composition and metabolism (114), as well as their innate and adaptive immune cell architecture (115). Hence, delineating the specific cellular pathways mediating the effects of relevant BA metabolites in the human colonic mucosa would be critical for gaining translatable insights into the aetiopathogenesis of IBD and identifying novel therapeutic strategies. Recent scientific advances provide a number of exciting avenues to further deconvolute the role of BAs in IBD in humans.

Firstly, the development of patient-derived intestinal organoids provide an unprecedented opportunity to investigate host-microbiota cross-talk, including metabolites such as BAs, in the human colonic mucosa (116). Intestinal organoids are 3D *ex vivo* structures derived from the human gut that are able to self-renew, self-organise, and recapitulate the architecture and function of the human gastrointestinal tract (117). Organoids can be derived from adult human intestinal stem cells (hASCs) or human induced pluripotent stem cells (iPSCs). The former gives rise to organoids consisting of intestinal epithelial cells only, whereas the latter generates organoids containing both epithelial and mesenchymal cells. Importantly, organoids derived from IBD patients have been found to retain disease-specific transcriptional and epigenetic signatures during uninflamed and inflamed states (118, 119). In addition, organoids can be co-cultured with various immune cell types to investigate immune-epithelial cross-talk. Other recent important developments include the generation of apical-out (or reverse polarity) organoids which allows easier access to the apical membrane of the intestinal epithelium for exposure studies (120), and the ability to “inflame” organoids through treatment with a cocktail of inflammatory mediators (121). In addition, recently it has been demonstrated that as expected human ileal and colonic epithelial organoids express higher levels of the *FXR* and bile salt transporter genes in comparison to duodenal epithelial organoids (122). One of the major gaps in knowledge is how BA receptor activation can influence the aforementioned IBD-relevant downstream pathways such as autophagy and the inflammasome pathway in the human intestinal mucosa. The advent of patient-derived organoid technologies will allow this gap to be addressed for the first time using a human *ex vivo* model of IBD.

Another interesting technology that could be utilised in the future is the bioengineering of the gut microbiota to modulate the BA pool. As mentioned above, dietary factors and microbial composition are important determinants of the bile acid pool. However, the specific bacterial strains and metabolic pathways required for the synthesis of particular BAs are not yet clearly understood. Recently, Funabashi et al. identified the complete metabolic pathway required to convert CA and CDCA into DCA and LCA and then engineered this pathway into *Clostridium sporogenes* (15). This pathway contains gene products from a bile-acid-induced operon (termed bai) comprising eight genes. This breakthrough provides the potential to alter the composition of the BA pool by engineering the microbiome. Such an approach could allow researchers to adjust luminal BA composition as desired to closely interrogate the effects of BAs in the gastrointestinal tract, as well as develop novel anti-inflammatory therapeutic strategies through modulation of the BA pool.

Finally, state-of-the-art bioinformatics approaches for modelling metabolomics data could provide deeper insights into BA signalling in IBD. One such approach that has been recently applied to the gut microbiome is genome-scale metabolic modelling. Genome-scale metabolic modelling evaluates the genome of organisms to identify genes that encode metabolic enzymes, which enables prediction of metabolic fluxes occurring in the metabolic pathways of an organism. Heinken et al. utilised genome-scale metabolic modelling and flux balance analysis to evaluate the differences in BA metabolism between IBD patients and healthy controls using metagenomic data (123, 124). Through this approach, they found that a single microbial species alone could not yield the entire repertoire of SBAs present in the gut, but microbial pairs could generate most of the BAs *in silico*. Their analysis identified species-specific bottlenecks in individual patients that limited PBA biotransformation to SBAs. Another exciting development is a novel resource for strain-specific metabolomics, which can help link metabolites to individual strains and host phenotype (125). These novel approaches could inform the development of precision editing strategies of the gut microbiota to ameliorate intestinal inflammation in IBD (126).

## Conclusion

BAs are an important class of microbial metabolites that are altered in IBD. Increasing evidence, largely from murine models, indicates that they influence inflammatory responses through cell type-specific actions on the gut mucosa. These may involve downstream effects on a number of critical cellular pathways that have been implicated in IBD including autophagy, apoptosis, and the inflammasome pathway. Further research is required to deconvolute BA signalling and their perturbations in IBD patients. Recent developments in human organoid technology and state-of-the-art bioinformatics approaches provide an exciting opportunity to better understand the role of BAs in IBD. This has potential to open the doors to novel therapeutic strategies such as precision editing of the gut microbiota.

## Author Contributions

JT wrote the first draft of the manuscript and reviewed/edited the article before submission. DM, SR, NP, and TK made substantial contributions to the discussion of content and reviewed/edited the article before submission.

## Funding

At the time of writing, JT was an Academic Clinical Fellow supported by the National Institute of Health Research (NIHR). DM and TK are supported by the Earlham Institute (Norwich, UK) in partnership with the Quadram Institute (Norwich, UK) and strategically supported by a UK Research and Innovation (UKRI) Biotechnological and Biosciences Research Council (BBSRC) Core Strategic Programme Grant for Genomes to Food Security (BB/CSP1720/1) and its constituent work packages, BBS/E/T/000PR9819 and BBS/E/T/000PR9817, as well as a BBSRC ISP grant for Gut Microbes and Health (BB/R012490/1) and its constituent projects, BBS/E/F/000PR10353 and BBS/E/F/000PR10355. NP and TK are supported by the Imperial NIHR Biomedical Research Centre (BRC).

## Conflict of Interest

The authors declare that the research was conducted in the absence of any commercial or financial relationships that could be construed as a potential conflict of interest.

## Publisher’s Note

All claims expressed in this article are solely those of the authors and do not necessarily represent those of their affiliated organizations, or those of the publisher, the editors and the reviewers. Any product that may be evaluated in this article, or claim that may be made by its manufacturer, is not guaranteed or endorsed by the publisher.
